# Genetic Variants of *Ehrlichia phagocytophila*^1^*,* Rhode Island and Connecticut

**DOI:** 10.3201/eid0805.010251

**Published:** 2002-05

**Authors:** Robert F. Massung, Michael J. Mauel, Jessica H. Owens, Nancy Allan, Joshua W. Courtney, Kirby C. Stafford, Thomas N. Mather

**Affiliations:** * Centers for Disease Control and Prevention, Atlanta, Georgia, USA; † University of Rhode Island, Kingston, Rhode Island, USA; ‡ Connecticut Agricultural Experiment Station, New Haven, Connecticut, USA

**Keywords:** *Ehrlichia phagocytophila*, ehrlichiosis, rickettsia, *Anaplasma phagocytophila*

## Abstract

Primers were used to amplify a 561-bp region of the 16S rRNA gene of *Ehrlichia phagocytophila* from *Ixodes scapularis* ticks and small mammals collected in Rhode Island and Connecticut. DNA sequences for all 50 *E. phagocytophila*-positive samples collected from 1996 through 1998 in southwestern Connecticut were identical to the sequence reported for *E*. *phagocytophila* DNA from confirmed human cases. In contrast, the sequences from 92 of 123 *E*. *phagocytophila*-positive Rhode Island samples collected from 1996 through 1999 included several variants differing by 1-2 nucleotides from that in the agent infecting humans. While 11.9% of 67 *E. phagocytophila*-positive ticks collected during 1997 in Rhode Island harbored ehrlichiae with sequences identical to that of the human agent, 79.1% had a variant sequence not previously described. The low incidence of human ehrlichiosis in Rhode Island may in part result from interference by these variant ehrlichiae with maintenance and transmission of the true agent of human disease.

Members of the genus *Ehrlichia* are obligate, intracellular bacteria in the order Rickettsiales. Although ehrlichial infections of veterinary importance were first described in 1935, the first case of human ehrlichiosis in the United States was reported in 1987 (1). The human pathogen was subsequently identified as *Ehrlichia chaffeensis* (2), and the number of reported human cases now exceeds 740 (3). In 1994, a second ehrlichial infection in humans was reported, called human granulocytic ehrlichiosis (HGE) because of its proclivity to infect neutrophils (4). Most HGE cases have been diagnosed in the northeastern and upper midwestern United States, although a few cases have been reported in Europe and northern California (5–12).

The close genetic and antigenic relationship of the HGE agent to two previously characterized species (*E. phagocytophila*, noted for infections of ruminants in Europe, and *E. equi*, the agent of equine granulocytic ehrlichiosis) has led to the suggestion that these three be classified as a single species, with *E. phagocytophila* as the precedent name. The 16S rRNA gene has been amplified and sequenced from confirmed human cases in both North America and Europe, and all sequences have been identical to the original published sequence for the HGE agent (4,8), with the exception of two cases recently reported from northern California that were the same as the *E. equi* 16S rRNA gene sequence (12). A variant that differed by 2 bp from the sequence of the HGE agent was reported in white-tailed deer in Maryland and Wisconsin and in *Ixodes scapularis* ticks collected in Rhode Island (13,14). Likewise, the 16S rRNA sequences determined from documented infections of horses and ruminants by various *E. phagocytophila* strains have differed by several bp from the sequences of the HGE agent. None of the variant forms have been shown to cause human disease. Another ehrlichia found in nature, which is closely related to *E. phagocytophila* but apparently does not cause human disease, is the white-tailed deer agent (15). Ehrlichiae closely related to *E. phagocytophila* recently have been identified in Colorado, where human ehrlichiosis is not endemic (16). These data suggest that only a subset of the *E. phagocytophila* strains that exist in nature can cause human disease. Although the HGE agent is now considered a member of the species *E. phagocytophila*, hereafter we will designate the isolates responsible for human disease as *E. phagocytophila*-human agent (EP-ha) to differentiate them from the 16S rRNA sequence variants we describe in this report.

Rhode Island and Connecticut, adjacent northeastern states, have similar frequency and distribution of vector *I. scapularis* ticks and primary reservoir hosts (white-footed mice and chipmunks) (17–19). However, the number of reported human infections with *E. phagocytophila* is dramatically higher in Connecticut than in Rhode Island. Through 1997, the average annual incidences (per one million population) of HGE in Connecticut and Rhode Island were 15.90 and 0.67, respectively (3). We investigated the frequency and distribution of *E. phagocytophila*, including EP-ha and *E. phagocytophila*-related variants, in potential reservoir and vector populations in Rhode Island and Connecticut.

## Materials and Methods

### Tick and Mammal Collections

Questing nymphal and adult black-legged ticks (*I. scapularis*) collected from four sites in South Kingston, Rhode Island, and one site in Bridgeport, Connecticut, were analyzed for the presence of granulocytic ehrlichiae (Figure). Collections of adult- or nymphal-stage ticks or both were available from ongoing tick surveillance conducted in each region from 1996 to 1999. Questing nymphal ticks collected from Bluff Point in southeastern Connecticut in 1997 were also available. The Rhode Island sites are all located in the state’s zone of highest *I. scapularis* density (17). Tick density was also high at the Bridgeport site. Ticks were collected by following standardized sampling procedures (17). All ticks were stored for <2 years in 70% ethanol until tested. Small rodents, including white-footed mice (*Peromyscus leucopus*) and chipmunks (*Tamias striatus*) live-trapped at the same locations were bled following procedures approved by the institutional animal care and use committees of each institution. Briefly, animals were trapped from July to September along transect lines or in trapping grids. An additional collection was made during May 1998 at the Bridgeport site. Blood was stored in EDTA at -80°C until tested for ehrlichiae by polymerase chain reaction (PCR) techniques and DNA sequencing.

**Figure:**
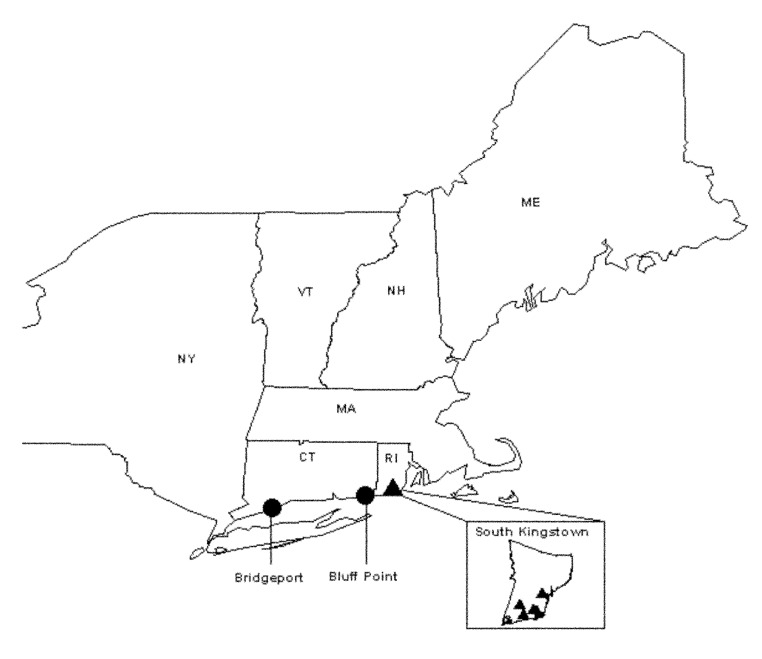
Map of northeastern United States, showing location of tick and rodent sampling sites.

### Sample Preparation

DNA was extracted directly from blood samples by using a QIAamp Blood Extraction Kit (Qiagen, Chatsworth, CA), according to manufacturer’s instructions. Briefly, detergent lysis was done in the presence of proteinase K for 10 min at 70°C. The lysed material was applied to a spin column containing a silica gel-based membrane and then washed twice. Purified DNA was eluted from the columns in 200 µL Tris-HCl (10 mM, pH 8.0) and stored at 4°C until used as template for PCR amplification. DNA was extracted from *I. scapularis* ticks by a modification of the manufacturer’s protocol for the QIAamp Tissue Kit (Qiagen) as described (13).

### PCR Analysis

A nested PCR that amplified a 546-bp portion from the 5´ region of the 16S rRNA gene was used to identify granulocytic ehrlichiae in tick and wildlife samples (13). Briefly, primary amplifications consisted of 40 cycles in a Perkin-Elmer 9600 thermal cycler (Perkin-Elmer, Applied Biosystems Division, Foster City, CA), with each cycle consisting of a 30-sec denaturation at 94°C, 30-sec annealing at 55°C, and a 1-min extension at 72°C. The 40 cycles were preceded by a 2-minute denaturation at 95°C and followed by a 5-minute extension at 72°C. Primary amplifications used primers ge3a and ge10r and reagents from the GeneAmp PCR Kit with AmpliTaq DNA Polymerase (Perkin-Elmer). Each reaction contained 5 µL of purified DNA as template in a total volume of 50 µL, as well as 200 µM each deoxynucleoside triphosphate (dNTP) (dATP, dCTP, dGTP, and dTTP), 1.25 units Taq polymerase, and 0.5 µM each of primer. Reaction products were subsequently maintained at 4°C until analyzed by agarose gel electrophoresis or used as template for nested reactions.

 Nested amplifications used primers ge9f and ge2 and 1 µL of the primary PCR product as template in a total volume of 50 µl. Each nested amplification contained 200 µM each dNTP (dATP, dCTP, dGTP, and dTTP), 1.25 units Taq polymerase, and 0.2 µM each of primer. Nested cycling conditions were as described for the primary amplification, except 30 cycles were used. Reactions were subsequently maintained at 4°C until analyzed by agarose gel electrophoresis or purified for DNA sequencing (13).

### DNA Sequencing and Data Analysis

 All samples producing positive PCR products were subjected to DNA sequencing reactions with fluorescent-labeled dideoxynucleotide technology (Dye Terminator Cycle Sequencing Ready Reaction Kit, Perkin-Elmer, Applied Biosystems Division). Sequencing reaction products were separated, and data were collected with an ABI 377 automated DNA sequencer (Perkin-Elmer, Applied Biosystems Division). The full sequence was determined for both strands of each DNA template to ensure maximum accuracy of the data. Sequences were edited and assembled with the Staden software programs (20) and analyzed with the Wisconsin Sequence Analysis Package (Genetics Computer Group, Madison, WI) (21).

## Results

Fifty (13.3%) of 375 *I. scapularis* ticks from Bridgeport, Conn., were PCR positive for ehrlichiae (Table 1). The percentage positive in each of the 4 years ranged from 6.1% in nymphs in 1998 to 23.3% in adults in 1996. Less year-to-year variation was noted in adult ticks, in which infection prevalence ranged from 11.7% (1997) to 23.3% (1996). PCR analysis of EDTA blood samples from white-footed mice collected in Connecticut during summer and fall 1997 and spring 1998 showed that 17 (36.2%) of 47 in 1997 and 3 (60%) of 5 in 1998 were positive (22). The amplification products were sequenced for each *Ehrlichia* PCR-positive mouse and tick. All products from samples collected in the Bridgeport, Conn., area from 1996 through 1998 had sequences identical to the 16S rRNA gene (EP-ha) previously amplified and sequenced from documented human infections in the Northeast and Upper Midwest United States and in Europe (4). The 16S rRNA sequence determined from adult ticks collected from Bridgeport in 1999 showed that all 12 positive samples also contained the human agent (EP-ha), although one of the ticks produced a mixed sequence, suggesting the presence of more than one agent. The PCR products from this tick were cloned, and individual clones were purified and sequenced. These data confirmed the presence of a mixed population of ehrlichiae containing some 16S rRNA sequences that matched EP-ha and some that differed from EP-ha by two nucleotides. The latter sequence was identical to a variant (called variant 1) previously described in ticks in Rhode Island and deer in Maryland and Wisconsin (13,14) (Table 2). In contrast to ticks and rodents from the Bridgeport area, nymphal ticks collected in 1997 from Bluff Point in southeastern Connecticut contained a nearly equal distribution of EP-ha (5 [55.6%] of 9 positives) and variant 1 (4 [44.4%] of 9 positives) ehrlichiae.

**Table 1:** Spatial and temporal variation in the occurrence and distribution of *Ehrlichia phagocytophila*-human agent (EP-ha) and *E. phagocytophila* variants, Connecticut and Rhode Island, 1996–1999

Collection site	Year	No.	Adult	Nymph	No. of PCR-positive ticks (%)		No. of PCR products sequenced	Proportion infected with—		
								EP-ha	Variant 1	Variant 2
Bridgeport, CT	1996	30	+		7	(23.3)	7	100%		
	1997	60	+		7	(11.7)	7	100%		
	1998	82		+	5	(6.1)	5	100%		
	1998	101	+		19	(18.8)	19	100%		
	1999	102	+		12	(11.8)	12	100%^a^	8.3%^a^	
Bluff Point, CT	1997	79		+	9	(11.4)	9	55.6%	44.4%	
South Kingstown, RI	1996	31	+		5	(16.1)	5	60%	40%	
	1997	112		+	52	(46.4)	30	10%	3.3%	86.7%
	1997	120	+		46	(38.3)	37	13.5%	13.5%	73%
	1998	91		+	5	(5.5)	5	20%	80%	
	1999	103		+	5	(4.9)	5	80%	20%	
	1999	81	+		10	(12.3)	10	80%	20%	

^a^Includes one tick that was positive for both the human granulocytic ehrlichiosis agent and variant 1. PCR = polymerase chain reaction.

**Table 2:** Variation within nucleotide region 74 to 446 in 16s rRNA gene sequences obtained for *Ehrlichia phagocytophila*-human agent (EP-ha),^a^ the Rhode Island variants, and *E. equi*

	Position number					
	76	84	157	176	284	299
EP-ha	A	G	A	G	C	A
RI variant 1	**G** ^b^	**A**	A	G	C	A
RI variant 2	A	G	A	**A**	**T**	A
RI variant 3	A	G	**G**	G	C	A
RI variant 4	A	G	A	G	C	**G**
*E. equi*/CA human	A	**A**	A	G	C	A

^a^ The number designations for the EP-ha 16S rDNA sequence correspond to those reported by Chen et al. (4). GenBank Accession No. U02521. ^b^ Variant base paris are shown in bold.-

Rhode Island samples from *I. scapularis* ticks, white-footed mice, and chipmunks contained *E. phagocytophila* variants as well as EP-ha. A total of 123 (22.9%) of 538 ticks were positive for *E. phagocytophila* by PCR, including 61 (26.3%) of 232 adults and 62 (20.3%) of 306 nymphs. DNA sequencing was performed on 92 of these PCR products, and overall, only 24 (26.1%) showed sequences identical to those of EP-ha. Fifteen (16.3%) ticks showed sequences corresponding to variant 1. The rest of the ticks (53 [57.6%]) had a novel sequence differing from EP-ha by 2 nucleotides and from variant 1 by 4 nucleotides (hereafter called variant 2) (Table 2).

PCR testing of blood samples from 19 Rhode Island chipmunks in 1996 detected 11 (57.9%) positives. DNA sequencing of these PCR products showed that nine were identical to the sequence of EP-ha; the remaining two represented novel variant sequences, each differing from EP-ha by a single nucleotide (variants 3 and 4; Table 2). Although both the white-tailed deer agent and the *E. equi*/CA human sequence variant (Table 2) are amplified by the PCR assay used in this study, neither agent has been detected in potential rodent reservoir populations in Connecticut or Rhode Island. Host and vector associations of EP-ha and the four *E. phagocytophila* variants found in Rhode Island are shown in Table 3.

**Table 3:** Host and vector associations of *Ehrlichia phagocytophila* and the four 16S rDNA sequence variants detected in Rhode Island,1996–1999

Host or tick species	EP-ha	Variant 1^a^	Variant 2	Variant 3	Variant 4
*Ixodes scapularis* ticks	+	+	+	–	–
White-footed mouse	+	–	+	–	–
Eastern chipmunk	+	–	–	+	+
White-tailed deer	–	+	–	–	–
Human^b^	+	–	–	–	–

^a^Variant 1 detected only in ticks in Rhode Island and Connecticut; positive deer samples were collected in Maryland and Wisconsin (13,14).  ^b^Based on samples from 35 confirmed human infections from various states, including Rhode Island and Connecticut.

The prevalence of *E. phagocytophila* in *I. scapularis* ticks (adults and nymphs combined, years 1996-1999) was higher in Rhode Island (22.8%) than in Bridgeport (13.3%) (p<0.001; Fisher’s exact test). This finding was also true for adult ticks: 26.3% were infected in Rhode Island compared with 15.4% in Bridgeport (p=0.002). Using either the total number of ticks tested or adult ticks only, the prevalence of *E. phagocytophila* in Rhode Island compared with Bridgeport was 1.7. However, if the 1997 Rhode Island data, which were skewed by an unusually large number of variants, are removed from the calculations, the percentage of *E. phagocytophila*-positive ticks (adults and nymphs) was significantly higher in Bridgeport (13.3%) than in Rhode Island (8.2%) (p=0.03). The same analysis, when restricted to the adult tick population, showed no significant difference between Connecticut (15.4%) and Rhode Island (13.4%) EP-positive ticks (p=0.6). If the 1997 Rhode Island data are excluded, the prevalence of *E. phagocytophila* in that state compared with Bridgeport was 0.6 for the total number of ticks tested and 0.8 for adult ticks only.

Infection prevalence data were available for adult and nymphal ticks from the same site for 3 years: Rhode Island in 1997 and 1999 and Bridgeport in 1998 (Table 1). For two of these, Rhode Island in 1999 and Bridgeport in 1998, the prevalence of *E. phagocytophila* was significantly higher (Rhode Island 1999; p=0.01) or borderline higher (Connecticut 1998; p=0.065) in adults than in nymphs. *E. phagocytophila* infection rates in nymphal and adult ticks from Rhode Island in 1997 did not differ significantly (p=0.21).

Temporal trends in tick infection rates showed that the prevalence of *E. phagocytophila* in Rhode Island nymphs was highest in 1997 and then declined in 1998 and 1999 (p<0.001; chi-square test for trend). However, the high number of variants found in both adult and nymphal ticks from Rhode Island in 1997 influenced this analysis, as *E. phagocytophila* prevalence in Rhode Island in 1997 was significantly higher than in all other years combined (p<0.001). In Bridgeport, no significant temporal trends were noted in *E. phagocytophila* infection rates in adult ticks (p=0.3), nor were significant prevalence or temporal trends noted in the rodents tested from any of the sites.

Analysis of the proportion of *E. phagocytophila*-positives that were variants showed that prevalence of the variants in Rhode Island (73.9% variants) was significantly higher than in Bridgeport (0.02% variants) (p<0.001). When the proportion of *E. phagocytophila*-positives that were variants was compared with the total number of positives for the two Connecticut sites, the ticks from Bluff Point (44.4% variants) showed significantly higher rates than ticks from Bridgeport (0.02% variants) (p<0.001).

## Discussion

Strains of *E. phagocytophila* found in nature are capable of causing disease in sheep, cattle, horses, dogs, cats, and humans. These strains, including the species previously known as the HGE agent and *E. equi*, are grouped as a single species on the basis of their close relationship at the genetic and antigenic levels. However, biological and ecological differences clearly exist between strains of *E. phagocytophila,* including varying host pathogenicity, vectors, DNA sequence, and geographic distribution. Small ribosomal subunit (16S) DNA sequences are highly conserved in bacteria and are often used to identify and differentiate bacterial species. The 16S rRNA gene sequences amplified from every confirmed human case, except for two isolated cases in northern California, have been identical to the *E. phagocytophila*-human agent (EP-ha) sequence determined by Chen et al. (4). PCR-positive white-footed mice (n=20) and *I. scapularis* ticks (n=38) collected in Bridgeport from 1996 through 1998 also harbored only *E. phagocytophila* identical in sequence to EP-ha for a 546-bp region of the 16S rRNA gene (4). Sequence analysis of PCR products from two Connecticut deer blood samples showed DNA identical to the EP-ha *p44* gene sequence (23). However, an *Ehrlichia* organism with a 16S rRNA gene sequence differing from EP-ha by a single nucleotide has been identified in white-tailed deer from Maryland and Wisconsin and in *I. scapularis* from Rhode Island (13,14).

In contrast to our results from Bridgeport, where we consistently found EP-ha, mice and ticks from Rhode Island had a significantly lower percentage of isolates identical to EP-ha, but several *E. phagocytophila* variants with novel 16S rRNA gene sequences. These data indicate that variant forms of *E. phagocytophila*, not yet associated with human or veterinary disease, frequently occur in Rhode Island. The same or additional *E. phagocytophila* variants may also occur in other regions of the United States, but this concept remains to be investigated.

Most PCR assays amplify products from the variant agents that are the same size as the EP-ha PCR product, so that variants are indistinguishable when the products are analyzed only by agarose gel electrophoresis. Therefore, results from other human-infection prevalence surveillance studies in ticks and rodents that have not included PCR product sequencing may be misleading. For example, if we had not sequenced our PCR products for 1997, we would have concluded that 46.4% of nymphal and 38.3% of adult *I. scapularis* ticks collected in southern Rhode Island were positive for EP-ha. Actually, only 11.9% of the positives that were sequenced and an estimated 5.0% of the total ticks tested were EP-ha positive, with the rest of the 1997 Rhode Island positives consisting of genetic variants not yet associated with human disease.

The 16S rRNA sequences obtained from tick and rodent samples collected from Bridgeport from 1996 through 1998 were identical to EP-ha. However, in 1999, one tick collected in that site was positive for both EP-ha and a variant (variant 1) previously found in Rhode Island. In a retrospective analysis of ticks collected at another eastern Connecticut site (Bluff Point) close to the Rhode Island border, variant 1 was found in 1997. Our inability to detect variant 1 despite extensive testing of samples collected in Bridgeport from 1996 to 1998 and its subsequent appearance in 1999 suggests that its geographic range may be expanding westward. Additional studies with larger sample sizes of ticks and rodents from Bridgeport and other locations in Connecticut are needed to assess the prevalence of EP-ha and the variants, as our Bridgeport results may not be representative of the entire state. In fact, the Bluff Point data suggest that other areas of Connecticut may have EP-ha/variant populations quite different from those in Bridgeport and more similar to the distribution noted in Rhode Island.

The identification of the coinfected tick in Connecticut represents the first detection of more than one strain of *E. phagocytophila* in a single tick vector in the United States, although the coinfection of *Ixodes ricinus* ticks by 2 *E. phagocytophila* strains has been reported in Europe (24). These data indicate that two strains of the agent are capable of coexisting in a single tick, at least transiently, and that they can survive the molting process, since the coinfection was found in an unfed, host-seeking adult tick.

The results from Rhode Island samples collected in 1997 are unusual in several regards. First, the rate of *E. phagocytophila*-positive ticks (42.2% nymphs and adults) was very high relative to all other tick populations sampled from 1996 through 1999, and many of the positives were variant 2 (79.1% of PCR-positives sequenced). Second, the 1997 Rhode Island ticks represent the only population in which *E. phagocytophila* prevalence was higher in nymphs than adults (46.4% nymphs and 38.3% adults). Finally, variant 2 sequences were also seen in samples collected in 1997 from white-footed mice and chipmunks but were not detected before and have not been detected after 1997. The fact that both nymphal and adult questing ticks were positive for variant 2 suggests that the variant was present in reservoir species during both larval and nymphal feedings and may have been present in reservoirs from late summer 1996 through summer 1997. Why this variant appeared only in Rhode Island during 1997, was the most prevalent strain infecting ticks that year, and then completely disappeared are matters of speculation. Variant 2 may be a more common infection in a reservoir species that we did not examine, which may be less commonly targeted by host-seeking ticks. Expression of variant 2 in the tick population could have resulted if, during 1996-1997, host populations preferred by immature *I. scapularis* (i.e., white-footed mice and chipmunks) were lower than normal, resulting in a higher proportion of ticks feeding on such atypical hosts harboring variant 2. After molting, nymphs infected as larvae the previous year could have transmitted variant 2 to the more preferred hosts of immature ticks, resulting in the variant 2-positive mice found in 1997. Subsequent reestablishment of normal host populations may then have diluted the prevalence of variant 2, as immature tick feeding reverted to the preferred hosts.

Although the function and biological importance of the genetic differences in *E. phagocytophila* strains are unknown, we hypothesize that the variants may be interfering with maintenance and transmission of the human disease-causing agent (EP-ha). Even if an increased human ehrlichiosis case surveillance effort in Connecticut is taken into account, the number of confirmed and suspected cases differs dramatically between the two neighboring states: several hundred cases were reported in Connecticut compared with fewer than 25 cases in Rhode Island during the same time period. From 1995 to 1997, 178 cases were confirmed or suspected (25), and case reports in Connecticut increased substantially in 1998 (228 provisional, 104 confirmed, Connecticut Dept. of Public Health). These adjacent states share many of the ecologic factors that support natural maintenance of both *Borrelia burgdorferi* and granulocytic ehrlichiae, such as populations of the tick vector *I. scapularis* and reservoir rodents, including white-footed mice (*P. leucopus*) (19,22). The incidence of Lyme disease in Connecticut and Rhode Island has been the highest in the nation for several years, with Connecticut having a reported incidence only approximately 1.3-1.5 times higher than Rhode Island’s (26). In contrast, through 1997 there was a 24-fold difference in the incidence of reported HGE cases in the two states (Connecticut 15.9; Rhode Island 0.67). Therefore, the *E. phagocytophila* variants may have a competitive advantage over the EP-ha, possibly in infecting certain reservoir or vector populations. A lower incidence of EP-ha and less human disease would therefore be expected in areas where the variants predominate, since a lower proportion of ticks would harbor EP-ha.

*Rickettsia rickettsii*, the etiologic agent of Rocky Mountain spotted fever, was first identified in the early 1900s on the basis of its association with human disease (27). Subsequent studies of veterinary infections and tick populations identified numerous *Rickettsia* species closely related to *R. rickettsii*, all clearly members of the spotted fever group but not associated with human disease. These species include *R. montana*, *R. rhiphicephali*, *R. parkeri*, *R. bellii*, and the “east side agent” *R. peacockii* (28,29). Nonpathogenic rickettsiae are thought to interfere with the development of more virulent *R. rickettsii* in *Dermacentor* ticks and may be found more often in ticks than are the more virulent species (30,31). Our data suggest that a similar situation may exist among the granulocytic ehrlichiae, with both pathogenic and nonpathogenic genetic variants coexisting in nature. Isolation of the new variants will allow us to address this competitive-advantage hypothesis experimentally in both ticks and mice through the use of mixed infections in the laboratory.

Identification and use of novel gene targets more variable than the 16S rRNA gene will eventually permit better assessment of variability between strains of *E. phagocytophila* (32–34). Future studies should include *E. phagocytophila* from additional geographic areas where a substantial number of human cases of granulocytic ehrlichiosis are reported (e.g., New York, Wisconsin, Minnesota) compared with areas (e.g., New Jersey, Pennsylvania, Delaware, Maryland, California) with similar vector densities but with little or no human disease.
